# Study on Damage Evaluation and Machinability of UD-CFRP for the Orthogonal Cutting Operation Using Scanning Acoustic Microscopy and the Finite Element Method

**DOI:** 10.3390/ma10020204

**Published:** 2017-02-20

**Authors:** Dongyao Wang, Xiaodong He, Zhonghai Xu, Weicheng Jiao, Fan Yang, Long Jiang, Linlin Li, Wenbo Liu, Rongguo Wang

**Affiliations:** 1 Center for Composite Materials and Structures, School of Astronautics, Harbin Institute of Technology, Harbin 150080, China; wangdongyao88@126.com (D.W.); hexd@hit.edu.cn (X.H.); xuzh@hit.edu.cn (Z.X.); xiaojiao458@163.com (W.J.); YngFan01@163.com (F.Y.); jianglong840@163.com (L.J.); lilinlin0310@163.com (L.L.); 2 School of Materials Science and Engineering, Harbin Institute of Technology, Harbin 150080, China

**Keywords:** damage, finite element method (FEM), polymer composite, scanning acoustic microscopy (SAM)

## Abstract

Owing to high specific strength and designability, unidirectional carbon fiber reinforced polymer (UD-CFRP) has been utilized in numerous fields to replace conventional metal materials. Post machining processes are always required for UD-CFRP to achieve dimensional tolerance and assembly specifications. Due to inhomogeneity and anisotropy, UD-CFRP differs greatly from metal materials in machining and failure mechanism. To improve the efficiency and avoid machining-induced damage, this paper undertook to study the correlations between cutting parameters, fiber orientation angle, cutting forces, and cutting-induced damage for UD-CFRP laminate. Scanning acoustic microscopy (SAM) was employed and one-/two-dimensional damage factors were then created to quantitatively characterize the damage of the laminate workpieces. According to the 3D Hashin’s criteria a numerical model was further proposed in terms of the finite element method (FEM). A good agreement between simulation and experimental results was validated for the prediction and structural optimization of the UD-CFRP.

## 1. Introduction

Unidirectional carbon fiber reinforced polymer (UD-CFRP) has been widely used in various fields including aviation, spaceflight, shipping, and structural engineering, owing to its superior performance of high specific strength, high specific stiffness, fatigue resistance, and damage tolerance [1,2,3]. Hence, conventional metal materials are gradually being replaced by UD-CFRP in the majority of modern industries and also in some traditional structural engineering fields [4]. Similar to metal materials, UD-CFRP assembly parts also inevitably need a consequent machining operation to achieve the desired geometric configuration; designed dimensional accuracy and mechanical joint requirement (such as rivet joint, bolted connection, etc.) [5], although UD-CFRP composites are generally fabricated in an almost net shape [6].

Unlike homogeneous materials, three major individual components of fiber, matrix, and fiber-matrix interface are incorporated in a UD-CFRP. These components have completely different microstructures and mechanical properties in micro-scale and macro-scale, respectively [7]. Due to heterogeneity and anisotropy, machining UD-CFRP encounters more difficulties including diverse cutting induced damages such as fiber pull-out, fiber bending, fiber matrix debonding, matrix crushing, matrix cracking, and delamination. Chip formation of UD-CFRP possesses the features of discontinuity and irregularity compared with conventional metal materials; more expensive tools, the polycrystalline diamond (PCD) tool, the PCD-coated tool or the cubic boron nitride (CBN) tool, are commonly utilized in machining UD-CFRP to replace the conventional high-speed-steel tool for mitigating tool wear [8]. To explore the machining mechanism of cutting UD-CFRP, an orthogonal cutting process is generally employed to simplify the machining operation, due to the fact that the machining operations such as turning, milling, drilling, and trimming can be considered as an orthogonal cutting process at the infinitesimal region where the tool impacts the workpiece instantaneously by ignoring the inclination of the main cutting edge of the tool.

To date, the experimental method and numerical simulation have both been carried out to reveal the machining mechanism and the chip formation of machining UD-CFRP. The experimental investigation on traditional machining of UD-CFRP was first performed by Koplev et al. [9] taking advantage of an orthogonal cutting test. It was considered that the fiber orientation angle of CFRP was the crucial factor in affecting chip formation and the cutting forces. Bhatnagar et al. [10] assessed the interactional relationship between the cutting forces and the fiber orientation angle in the orthogonal cutting of UD-CFRP. Furthermore, a predictive model referring to the frictional conditions and tool geometric parameters was proposed to forecast the chip formation and cutting force. Wang and Zhang [11] found that the final depth of cut differed from the nominal depth of cut in the test of cutting UD-CFRP, because of the “bouncing back” phenomenon. The fiber orientation angle was considered the most influential factor that determined the surface integrity, and 90° was the critical angle. Aiming at the damage failures, Wang and Ramulu [6] considered that the variation of fiber orientation angles generated three cutting mechanism: (1) in 0° fiber orientation, fractures occurred along the interface and were induced by cantilever bending of fibers; (2) in positive fiber orientations up to 75°, fractures were induced by compression and grew in a vertical direction to the fiber axis; (3) more than 90°, both in and out of plane shear fracture led to severe deformation. Besides, the relationship between tool geometry, cutting parameter and cutting forces were also explained. Except for cutting force and fiber orientation, surface quality was also a research emphasis for studying the machining mechanism and optimizing the cutting parameters of machining UD-CFRP. Microscopic analysis and surface roughness were generally utilized to characterize the surface quality. In the literature [3,12,13,14,15], feed rate was found be a key factor in affecting the surface finish in machining UD-CFRP.

With the development of high-performance computers, numerical simulation can significantly reduce the experimental cost and significantly enhance efficiency. Therefore, the finite element method (FEM) has played a most important role in analyzing cutting forces, fracture mechanisms, and sub-surface damage in machining UD-CFRP composites. In recent years, numerous two-dimensional (2D) and three-dimensional (3D) orthogonal cutting models were developed to simulate the machining operation of UD-CFRP. Two dimensional equivalent homogeneous anisotropic material (EHAM) models were developed by Arola and Ramulu [16] and Mahdi and Zhang [17] to study the fracture and material removal in orthogonal cutting of CFRP. The numerical simulations indicated that the predicted forces were in good agreement with the experimental results. Nayak et al. [18] created a two-phase (single fiber and matrix) model on the micro-scale to investigate the sub-surface damage (matrix cracking and fiber-matrix debonding) during a cutting process. Values of the cutting force were obtained from the simulation under the conditions of various fiber orientations and the final results were validated by experimental observation. However, the predicted thrust force did not agree well with the experimental data. Venu Gopala Rao et al. [19] used the cohesive zone model (CZM) to simulate a fiber–matrix interface in an equivalent homogeneous material (EHM) model of an orthogonal cutting of UD-composites. Matrix damage, fiber-matrix debonding, fiber fractureand cutting forces were all represented in the micro mechanical model for a better understanding of machining mechanism and material removal. It was also found that fiber orientation angles dominated the sub-surface damage, fiber failure mode (crushing or tensile failure) and the chip formation mechanism. Lasri et al. [20] investigated a finite model based on three sets of failure criterions involving Hashin, Maximum stress, and Hoffman criteria to simulate the sub-surface damage and forces per unit width in an initial segment of the orthogonal cutting process. The finite model with Hashin failure criterion was indicated to have better agreement with the experimental data. To analyze the influence of numerical parameters (mesh size, mesh orientation) and tool geometry, Soldani et al. [21] developed a 2D numerical model with the Hashin criterion for cutting CFRP. It was demonstrated that the chip morphology and cutting induced damage were extremely sensitive to numerical parameters and tool geometry. In the research of Santiuste et al. [22] a 2D plane stress model and a 3D model which had cohesive element interfaces were implemented in a comparative analysis through a vectorized user material subroutine (VUMAT). It was derived that the 3D model had advantages in simulating the cutting damage zone and delamination in orthogonal cutting. Besides, compared to a 3D model, a 2D model had a lower fidelity while the width of the model became thicker.

In previous studies of the orthogonal cutting process of UD-CFRP, both experimental test and numerical simulation were rarely concentrated on quantitative characterization of cutting induced damage in machining UD-CFRP, although the damage is a most significant factor in generating structural failure and reducing fatigue life for composite products. This paper is mainly focused on measuring cutting forces (cutting force and thrust force) and representing cutting induced damage (sub-surface damage) in an orthogonal cutting process of UD-CFRP sheets with various machining parameters and fiber orientation angles. By using scanning acoustic microscopy and digital image analysis (DIA), the cutting induced damages of UD-CFRP sheets are quantitatively characterized. In addition, a 3D EHAM finite element model of an orthogonal cutting process is proposed and validated by the experimental results. This model is used to optimize the machining parameters for the control of cutting induced damage.

## 2. Experimental Details

### 2.1. Fabrication of CFRP Laminates

T700/TDE-85 CFRP laminates were fabricated by means of the filament winding technique. The CFRP laminates were cured at 120 °C with a dwell time of 12 h at a heating rate of 1 °C/min. The final CFRP laminates were incorporated of 16 layers of plies and with a thickness of 2 mm. The volume fraction of carbon fiber in the CFRP laminate was 59.7%. The mechanical properties of T700/TDE-85 unidirectional laminates are collated in Table 1.

**Figure 1:**
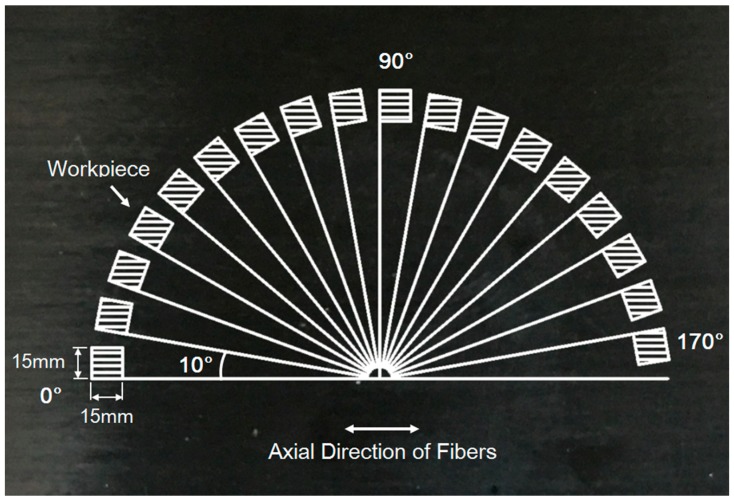
Definition of fiber orientations from 0° to 170° and geometric dimensions for unidirectional carbon fiber reinforced polymer (UD-CFRP) workpiece sheets.

**Figure 2:**
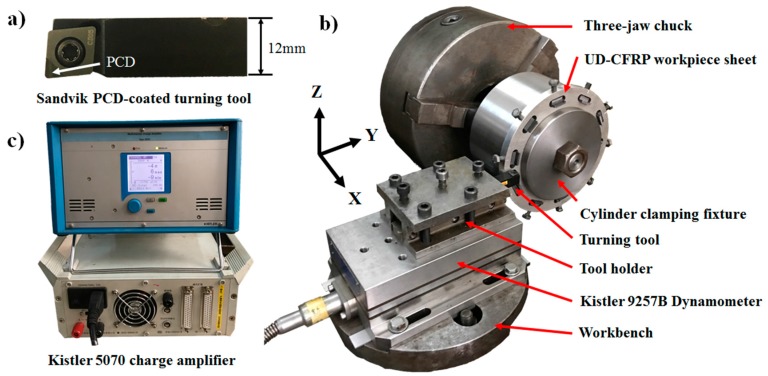
(**a**) Polycrystalline diamond (PCD)-coated turning tool; (**b**) Experimental setup of orthogonal cutting test of UD-CFRP workpieces; (**c**) Kistler 5070 multichannel charge amplifier.

In this study, a vertical sawing machine was employed to cut the unidirectional CFRP laminates into a designed dimension of 15 mm × 15 mm × 2 mm. The workpiece sheets with fiber orientation angles (FOA) of 0°, 10°, 20°, 30°, 40°, 45°, 50°, 60°, 70°, 80°, 90°, 100°, 110°, 120°, 130°, 135°, 140°, 150°, 160°, and 170° were obtained from the UD-CFRP laminates by clockwise rotating the cutting direction 10° (or 5° for the sheets with fiber orientation of 45° and 135°). The schematic illustration of fiber orientation is shown in Figure 1. A PCD-coated turning tool (CoroTurn^®^107, Sandvik Coromant Company, Fair Lawn, NJ, USA) was introduced and had a rake angle of 5° and a clearance angle of 7° (Figure 2a) in the experiments.

### 2.2. Experimental Setup and Parameters 

The orthogonal cutting experiment of UD-CFRP workpieces was conducted on a horizontal lathe (CA6140, Shenyang Machine Tool Co. Ltd., Shenyang, China) commonly used for a turning process. A cylinder clamping fixture with a diameter of 160 mm was equipped on the three-jaw chuck of the lathe. The cylinder clamping fixture was incorporated of ten rectangular grooves (depth of 10 mm, width of 8 mm, and length of 15 mm) to hold the workpiece sheets perpendicularly to its upper surface so that the orthogonal cutting test could be achieved by a turning process. According to the sequence of fiber orientation angles, UD-CFRP workpiece sheets were successively inserted into the grooves of the cylinder clamping fixture, and fastened by bolts and steel shims, as shown in Figure 2b. A quartz three-component dynamometer (Type 9257B, Kistler, Winterthur, Switzerland) used in the investigation was connected to a multichannel charge amplifier (Type 5070, Kistler) and mounted by a toolholder with an installed turning tool to measure the values of the cutting force and the thrust force of the orthogonal cutting experiment. The charge signals were transformed into values of instantaneous cutting forces in three dimensions and shown by a dynamic force acquisition system in the computer. The dynamometer provided a high sensitivity of 3.742 pC/N in each dimension and a sampling frequency of 5000 Hz.

**Figure 3:**
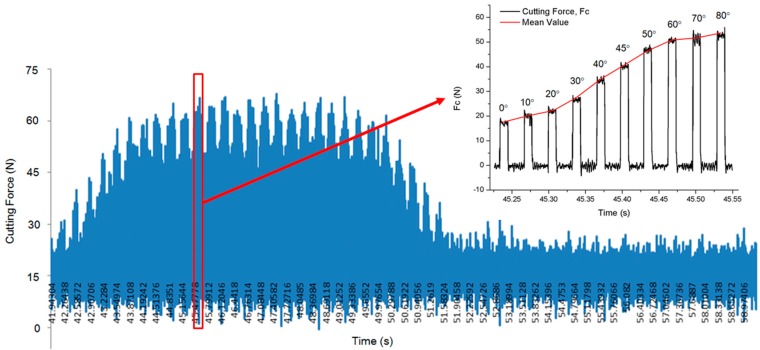
Cutting force data from an orthogonal cutting test of UD-CFRP specimen with a cutting speed of 309.5 m/min, a cutting depth of 0.2 mm, and fiber orientation angles from 0° to 80°.

Due to the experimental setup of the turning process, forces of three mutually perpendicular directions were defined as thrust force, feed force, and cutting force in the X-axis, Y-axis, and Z-axis of the dynamometer, respectively. While the feed rate of the turning process was 12 mm/min which was negligible compared with the cutting speed in the test, the turning of CFRP workpiece sheets could be simplified as an orthogonal cutting process. Since the forces of ten workpieces with sequential fiber orientation angles could be achieved in a single turning process, the waveform of the orthogonal cutting test was able to be approximated to a square wave form with a periodicity, as shown in Figure 3. The first experimental sheet should be labeled before the cutting process started in order to distinguish the forces from the data. A single-factor experiment was carried out, and the cutting parameters restricted by the horizontal lathe through all the experiments as shown in Table 2.

### 2.3. Damage Analysis with Scanning Acoustic Microscopy 

After post machining treatments, uncertain cutting induced damages occur in the machined region of a UD-CFRP workpiece. In the literature, it can be found that cutting induced damage can reduce the quality of the machined surface and the dimensional precision of machined UD-CFRP; meanwhile, the cutting induced damage weakens the residual stiffness of UD-CFRP and the fatigue resistance performance of UD-CFRP. Commonly, these sub-surface damages and defects cannot be detected intuitively. Thus, a non-destructive evaluation (NDE) technique is utilized to characterize the level of sub-surface damages using scanning acoustic microscopy (SAM) analysis, owing to the acoustic impedance performance and the capability of ultrasonic absorption and reflection for various materials. SAM has outstanding advantages in failure analysis, materials analysis, and performance characterization: (1) SAM can provide a variety of imaging modes, and has a function of measuring a pixel pitch; (2) it has an ultra-high frequency transducer and a high image resolution of 7 microns; (3) SAM possesses a high robustness in a scanning process [23,24,25,26,27]. 

**Figure 4:**
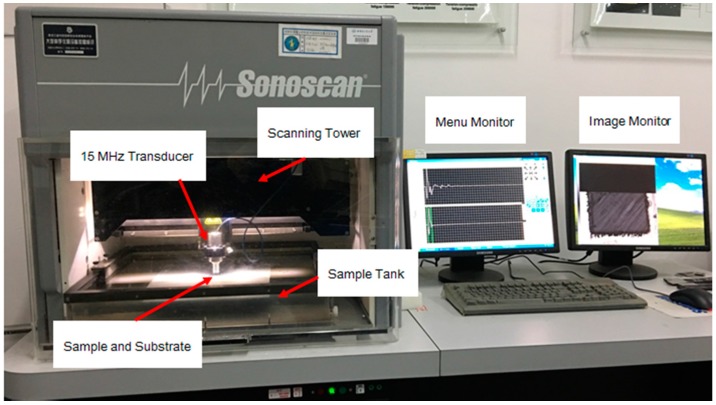
Major Components of the Sonoscan^®^ D9500^TM^ scanning acoustic microscopy.

**Figure 5:**
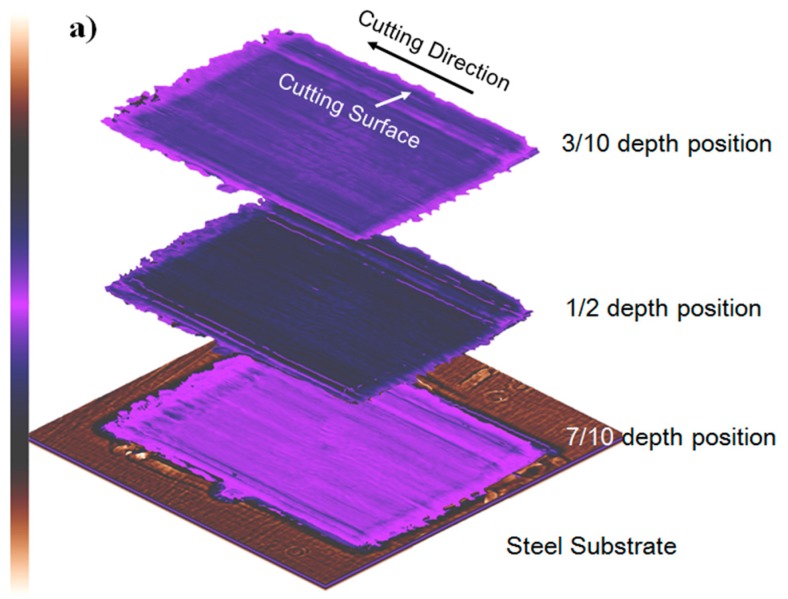

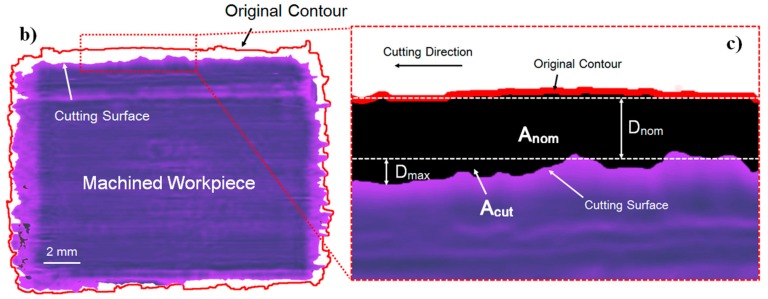
(**a**) Scanning acoustic microscopy (SAM) scanning images at three different positions of one UD-CFRP workpiece with a fiber orientation of 0°; (**b**) A schematic of the original contour of an unprocessed workpiece obtained by SAM and the comparison between the original contour and the scanning image of machined workpiece for one unique workpiece; (**c**) Expressions of the variables used in damage factors.

In this study, a scanning acoustic microscopy (CSAM^®^ D9500™) manufactured by Sonoscan^®^ (Cook county, IL, USA, shown in Figure 4) was employed to characterize the cutting induced damage of a machined UD-CFRP workpiece. The scanning was performed in water medium with a 15 MHz transducer. The scanning parameters were set as follows: the scanning area was 18.40 mm × 18.40 mm covered by 2048 pixels, the scanning speed was at a low level of 50.8 mm/s and the scanning time of a single sample was 14 min and 55 s. Before the orthogonal cutting experiment, all UD-CFRP sheets were tested by SAM to eliminate the experimental workpieces with high porosity and defects, and record the original contours (shown in Figure 5b) for quantification of UD-CFRP damage.

### 2.4. Damage Determination

In the studies of drilling CFRP, damage factors were created to determine the level of delamination quantitatively [5]. These damage factors were summarized into three categories as one-dimensional factors, two-dimensional factors, and combined factors [23,28]. For assessing the extent of sub-surface damage of the orthogonal cutting process, the depth of damage was used in the finite simulations modeled by Lasri et al. [20], Zenia et al. [1], and Bhatnagar et al. [29].

Being similar to damage factors for delamination quantification and depth of sub-surface damage, a one-dimensional factor F_dep_ and a two-dimensional factor F_a_ were established to analyze the cutting induced damage in the orthogonal cutting test. These two expressions of damage factors are shown in Table 3, where D_max_ is the maximum depth of cutting induced damage, D_nom_ is the nominal depth of cut, A_cut_ is the whole damaged area resulting from cutting process, and A_nom_ is the damaged area with the nominal depth of cut. Digital Image Analysis (DIA) was implemented by a commercial computing software Matlab 8.3 [30] developed by the MathWorks, Inc. to capture the changes of D_max_, D_nom_, A_cut_, and A_nom_ from the SAM scanning images. Figure 5b presents three scanning images of a machined 0° UD-CFRP workpiece sheet in 3/10, 1/2, and 7/10 depth position, respectively. Figure 5c expresses the definitions of the variables of damage factors: D_max_ is the maximum depth of the black area, D_nom_ is the vertical distance between two white dashed lines, A_cut_ is the area filled with black, and A_nom_ is the area within the white dashed lines.

## 3. Numerical Modeling of Orthogonal Cutting in UD-CFRP

The finite element method (FEM) has played a significant role in researching of cutting forces, initiation of damage, damage propagation, and failure modes for machining UD-CFRP, since the work of Chang F. K. and Chang K. Y. [31]. Through credible numerical models on micro- or macro-scale and reasonable damage criterion implemented by a user subroutine, fracture modes, the residual stiffness and the internal integrity of a machined workpiece which is difficult to capture in an orthogonal cutting experiment, can be achieved. Here, a 3D numerical solid element model assisted by a vectorized user material subroutine (VUMAT) for an orthogonal cutting process was developed and employed to predict cutting forces and characterize cutting induced damage. A comparative analysis between experimental and numerical data was carried out to validate the numerical model.

### 3.1. Description of the Basic Model

**Figure 6:**
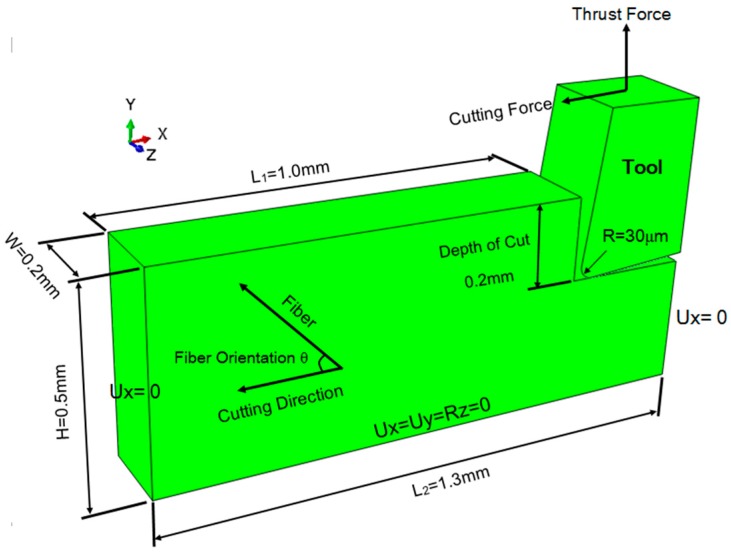
Modeling and boundary conditions of an orthogonal cutting process of a UD-CFRP workpiece.

A 3D meso-scale equivalent homogeneous anisotropic model of cutting UD-CFRP was developed using a commercial FEA code ABAQUS 6.10 [32]. As shown in Figure 6, the UD-CFRP workpiece with a pre-cut step that could lead to a steady penetrating process for contacted elements of the tool and the workpiece had an approximate geometric dimension of 1.3 mm × 0.5 mm × 0.2 mm (length × height × width). The material properties of UD-CFRP used in the simulation are listed in Table 1. The displacements of the workpiece bottom were constrained completely in three directions (X, Y, and Z), and both sides in the left and the right-hand of the workpiece were constrained in the horizontal direction (parallel to the direction of cutting speed). UD-CFRP workpiece and the PCD cutting tool were all finely meshed about 10 μm using 3D solid elements (C3D8R elements) which are eight-noded, linear hexahedral elements with reduced integration, hourglass control, and element deletion in order to precisely present the chip formation and the extent of cutting induced damage.

The numerical cutting tool was modeled as the one used in the experiment with a rake angle of 5°, a clearance angle of 7° and a nose radius of 0.03 mm. In the simulation of cutting UD-CFRP, the tool was set as a rigid body with the following points: (1) the deformation of the tool was to be neglected, since the moduli of PCD and CFRP in the fiber direction were 860 and 138 GPa, respectively; (2) the stress-strain, friction, and tool wear of the cutting tool were not the focus in this study; (3) computational cost could be economized for simulating this severe nonlinear large-deformation process. A reference point was set at the middle of the tool nose, binding the whole elements of the tool. The load parameter, a constant cutting velocity, used in the experimental work was attached to the reference point to control the orthogonal cutting operation. 

In the finite element analysis (FEA) of machining UD-CFRP, especially drilling UD-CFRP, cohesive elements were generally employed to model the fiber-matrix interface due to the advantage of predicting delamination. However, delamination was only one of the various failure modes and induced minor influence on the sub-surface damage in the orthogonal cutting process because the mechanical stress through the thickness did not need to focus on an orthogonal cutting process. Moreover, cohesive elements induced excessive element distortion and the time for simulating the cutting process of UD-CFRP could be significantly increased by using the cohesive elements. Ultimately, a 3D equivalent homogeneous anisotropic material (EHAM) model was developed to demonstrate the damage propagation to determine the cutting forces.

A dynamic-explicit analyzing step with a time period of 0.0001 s, a linear bulk viscosity parameter of 0.05 and a quadratic bulk viscosity parameter of 0.1 was constructed. To avoid the element penetration phenomenon, an interaction of surface-to-surface contact was created, with the first surface of both rake face and flank face of the cutting tool and the second surface of the node region of the machined UD-CFRP workpiece. Mechanical constraint formulation of kinetic contact method and a finite sliding formulation were used to control the behavior of contact. As shown in the literature [1,33], the friction coefficient of the tool and UD-CFRP had an outstanding effect on cutting forces and damage modes in machining UD-CFRP and a constant friction coefficient of 0.4 abiding by the Coulomb friction law was suggested.

### 3.2. Failure Criterion and Element Deletion

The failure criterion of UD-CFRP composites was the main theoretical basis for implementing numerical simulation of the orthogonal cutting operation and chip formation. Meanwhile, the failure criterion had a significant effect on the results of damage modes and cutting forces in the orthogonal cutting simulation. Since the analytic method was carried out to reveal the failure mechanism of UD-CFRP, a variety of typical failure criteria were proposed. These failure criteria of UD-CFRP could be classified into two categories: (1) aiming at the anisotropy, damage was estimated to occur when an external stress reached the ultimate strength in a certain direction of the material, such as Tsai-Wu failure criteria, etc.; (2) aiming at the heterogeneity, initial damage was determined by the failure modes of fibers, matrices, and interlaminations, such as Hashin, Chang-Chang, Puck, and Hou failure criteria, etc. [31,34,35,36,37,38,39,40,41]. Among these failure criteria, Hashin’s criteria are well known for their steady performance and therefore commonly used.

The Hashin’s criteria are represented as follows:

Tensile fiber failure for $\sigma_{1}\geq 0$

(3) $$F_{f}^{T}={\left(\frac{\sigma_{1}}{X_{T}}\right)}^{2}+\frac{\tau_{12}^{2}+\tau_{13}^{2}}{S_{12}^{2}}=\{\begin{array}{c} \;d_{f}^{T}\geq 1\;\;\;\;\;\;\;\;failure\\ \;d_{f}^{T}<1\;\;no\;failure \end{array}$$

Compressive fiber failure for $\sigma_{1}<0$

(4) $$F_{f}^{C}={\left(\frac{\sigma_{1}}{X_{C}}\right)}^{2}=\{\begin{array}{c} \;d_{f}^{C}\geq 1\;\;\;\;\;\;\;\;failure\\ \;d_{f}^{C}<1\;\;no\;failure \end{array}$$

Tensile matrix failure for $\sigma_{2}+\sigma_{3}\geq 0$

(5) $$F_{m}^{T}=\frac{{\left(\sigma_{2}+\sigma_{3}\right)}^{2}}{Y_{T}^{2}}+\frac{\tau_{12}^{2}-\left(\sigma_{2}\times \sigma_{3}\right)}{S_{23}^{2}}+\frac{\tau_{12}^{2}-\tau_{13}^{2}}{S_{13}^{2}}=\{\begin{array}{c} \;d_{m}^{T}\geq 1\;\;\;\;\;\;\;\;failure\\ \;d_{m}^{T}<1\;\;no\;failure \end{array}$$

Compressive matrix failure for $\sigma_{2}+\sigma_{3}<0$

(6) $$F_{m}^{C}=\frac{\left(\sigma_{2}+\sigma_{3}\right)}{Y_{C}}\left[{\left(\frac{Y_{C}}{2S_{23}}\right)}^{2}-1\right]+{\left(\frac{\sigma_{2}+\sigma_{3}}{2S_{23}}\right)}^{2}+\frac{\left(\tau_{23}^{2}-\left[\sigma_{2}\times \sigma_{3}\right]\right)}{S_{23}^{2}}+\frac{\left(\tau_{12}^{2}+\tau_{13}^{2}\right)}{S_{12}^{2}}=\{\begin{array}{c} \;d_{m}^{C}\geq 1\;\;\;\;\;\;\;\;failure\\ \;d_{m}^{C}<1\;\;no\;failure \end{array}$$

where $\sigma_{1}$, $\sigma_{2}$, $\sigma_{3}$, $\tau_{12}$, $\tau_{13,}$ and $\tau_{23}$ are the components of the stress tensors; $X_{T}$ and $Y_{T}$ are the tensile strengths in 1-direction (the fiber direction) and 2-direction (transverse to the fiber direction), respectively; $X_{C}$ and $Y_{C}$ are the compression strengths in respective directions; $S_{12}$, $S_{13}$, $S_{23}$ are the shear strengths in respective principal planes. $d_{f}^{T}$, $d_{f}^{C}$, $d_{m}^{T},$ and $d_{m}^{C}$ are damage variables concerning the failure modes from the Hashin’s criteria, and there is a damage variable d that is assumed to be the maximum of $d_{f}^{T}$, $d_{f}^{C}$, $d_{m}^{T},$ and $d_{m}^{C}$ to control the damage contours in visualization of ABAQUS. 

In the FEA software ABAQUS, only 2D Hashin’s criteria of shell elements are provided. Therefore, to implement the 3D orthogonal cutting process, the 3D Hashin’s criteria of solid elements are required to employ in the ABAQUS/Explicit with a user defined subroutine. On the other hand, to simulate the chip formation of UD-CFRP, an element deletion procedure is needed to embed into the user defined subroutine. The function of element deletion is deemed to be a crucial way to conquer the disadvantages of FEA, because FEM is based on continuum mechanics. In the theory of continuum mechanics, research objects are assumed to possess the characteristic of continuity in space. Under such a theoretical hypothesis the elements in FEM will never vanish or suffer failure in a machining simulation operation, although the chips formed in a machining process are actually existent. Thus, in order to afford the ABAQUS capacity of simulating the chip formation and damage propagation, a user defined subroutine (VUMAT for ABAQUS/Explicit) is indispensable. 

In the algorithm of VUMAT used in this paper: (1) first, material properties were read and terms of stiffness matrix were computed; (2) second, a characteristic of purely elastic material was assumed and total strains were updated; (3) third, failure of materials was evaluated by 3D Hashin’s criteria, the damaged stiffness matrix and stresses were recomputed when a new damage occurred; (4) finally, a new damage variable d which was the maximum of $d_{f}^{T}$, $d_{f}^{C}$, $d_{m}^{T},$ and $d_{m}^{C}$ and calculated from failure criteria at the integration point of the elements, was utilized to determine the initial element deletion when d was equal to 1. After an element deletion procedure, the element lost its total stiffness, was removed from the mesh, and provided no effect on the following simulating steps. 

The 3D Hashin’s criteria VUMAT with a function of element deletion was employed in the numerical orthogonal cutting model in Section 3.1. Cutting induced deleted elements in a single simulation process were captured with a python script so that the damage factors F_dep_ and F_a_ defined in Section 2.4 could be achieved to quantitatively characterize the extent of cutting damages. 

## 4. Results and Discussion

### 4.1. Results of Orthogonal Cutting Experiments

#### 4.1.1. Effects of Cutting Parameters and FOA on Cutting Force and Thrust Force

**Figure 7:**
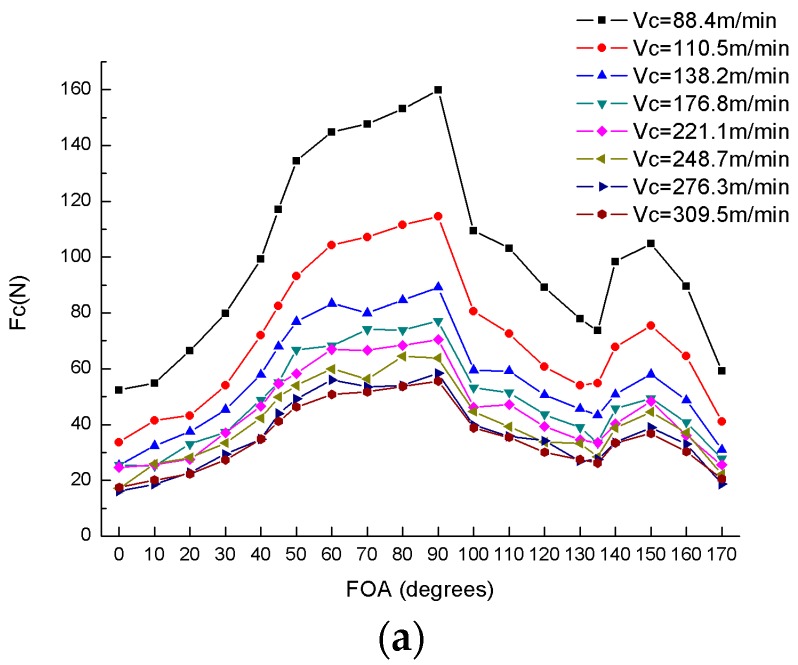

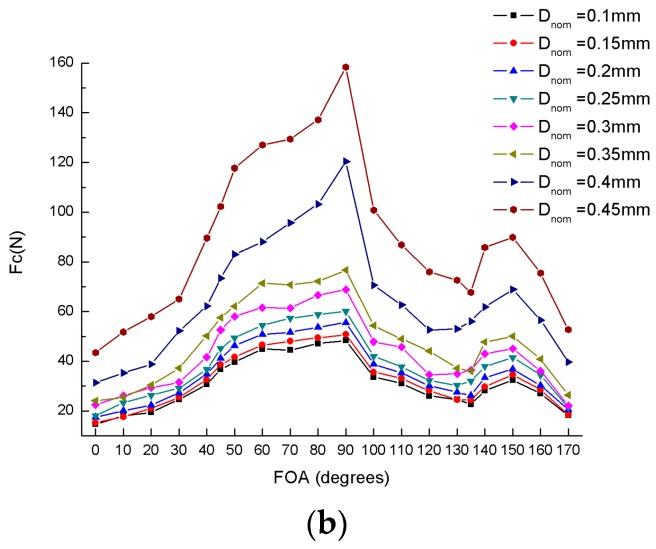
Variation of cutting forces (F_c_) versus fiber orientation angles (FOA): (**a**) Varying cutting speed V_c_; (**b**) Varying nominal depth of cut D_nom_.

Figure 7a illustrates the variation of the cutting force with respect to the fiber orientation angle under the condition of varying cutting speed and a constant cutting depth of 0.2 mm. It can be seen that the value of the cutting force improved remarkably with an increase in the cutting speed. Figure 7b shows the variation of the cutting force versus fiber orientation angle with a condition of a varying depth of cut and a constant cutting speed of 309.5 m/min. Accompanying the increase of the nominal depth of cut, the cutting force begins to decrease sharply and then descend smoothly. According to the Figure 7, the variation of the cutting force can be divided into three major categories: (1) in the range of 0°–90°, the cutting force almost linearly increases and reaches the maximum critical point of 90° with the rise of the fiber orientation angle; (2) in the range of 90°–135°, with the growth of the fiber orientation angle the cutting force shows a rapidly decreasing trend till it meets the minimum value at 135°; (3) in the range of 135°–180°, the variation curve of the cutting force is considered to be a parabola with the maximum at around 150° that is much smaller than 90°.

**Figure 8:**
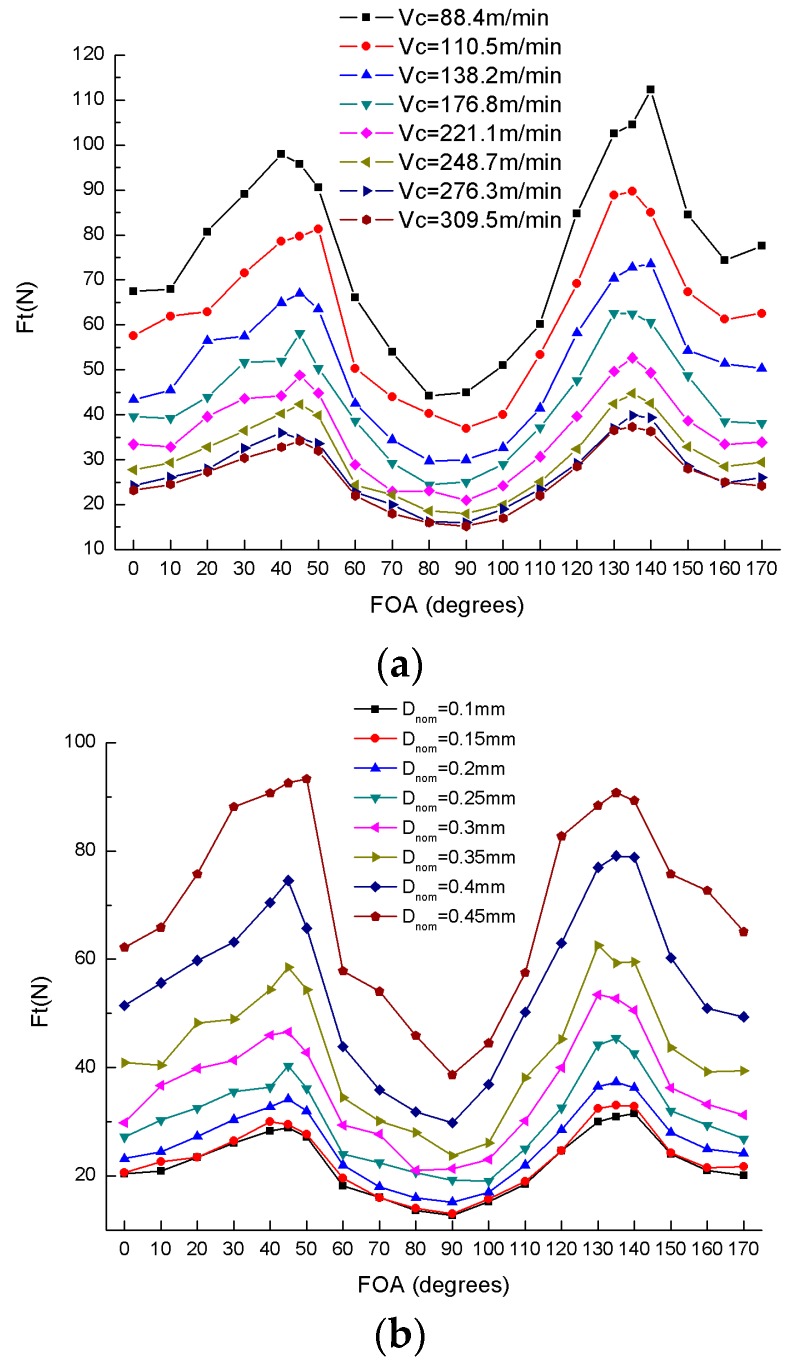
Variation of thrust forces (F_t_) versus fiber orientation angles (FOA): (**a**) Varying cutting speed V_c_; (**b**) Varying nominal depth of cut D_nom_.

Likewise, the variation of thrust force is presented in Figure 8. Similar to the cutting force, the thrust force is proportional to the depth of cut and inversely proportional to the cutting speed. Unlike the cutting force, the thrust force can be only divided into two parts: (1) 0°–90°; and (2) 90°–180° due to the crucial point of 90°. In both parts, the thrust force initially increases and then decreases, and achieves the maximum value at 135° (the value at 45° is almost close to 135°) and the minimum value at 90°.

During an orthogonal cutting experiment, the fiber orientation angle of UD-CFRP laminates has a decisive influence on the cutting force and the thrust force; the fiber orientation angle of 90° can be considered as a critical angle; and the thrust force is generally less than the cutting force, which is similar to the experimental results observed by Soldani et al. [21] and Nayak et al. [18].

#### 4.1.2. Evaluation of Damage Factors

**Figure 9:**
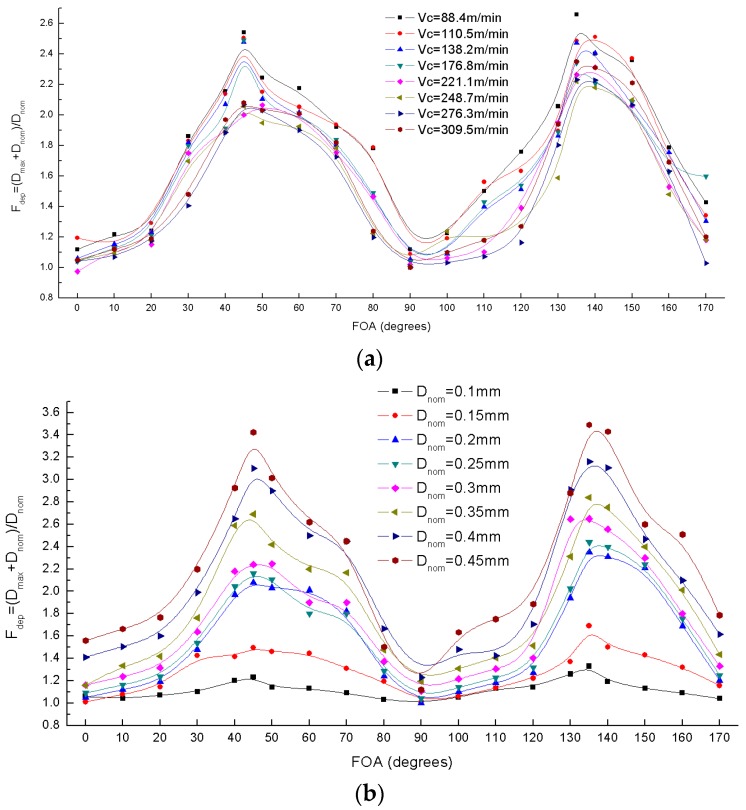
Variation of damage factor F_dep_ versus fiber orientation angles (FOA): (**a**) Varying cutting speed V_c_; (**b**) Varying nominal depth of cut D_nom_.

**Figure 10:**
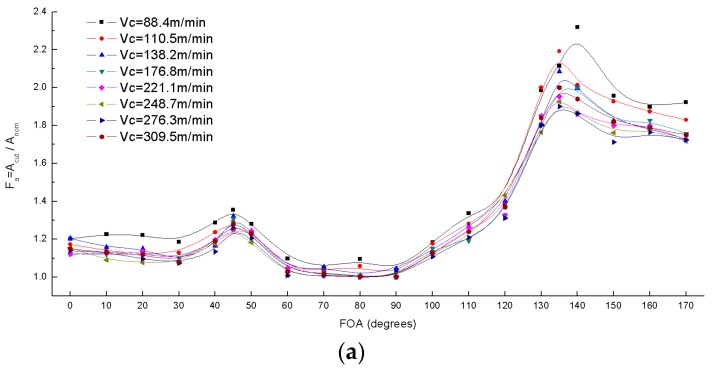

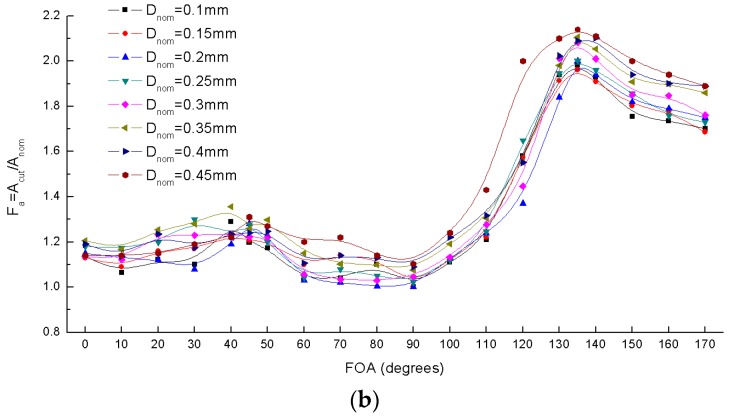
Variation of damage factor F_a_ versus fiber orientation angles (FOA): (**a**) Varying cutting speed V_c_; (**b**) Varying nominal depth of cut D_nom_.

As shown in Figure 5, the scanning images of one UD-CFRP workpiece were acquired at three different depth positions through the thickness in order to reduce the experiment error. 1-D damage factor F_dep_ and 2-D damage factor F_a_ were calculated from Equations (1) and (2). The results are presented in Figure 9 and Figure 10.

Figure 9 shows the effect of the fiber orientation angle on the damage factor F_dep_ with the variation of the cutting speed and the nominal depth of cut. Comparing with the cutting speed, the effect of the depth of cut on F_dep_ is more severe, though they are both proportional to F_dep_. At low levels of the depth of cut, F_dep_ remains within a range of 1–1.2, indicating that UD-CFRP has just minimal damages with a small cutting depth. The damage factor F_dep_ reaches the maximum at 45° or 135°, and the minimum value at 90° which is almost equal to 1 under various cutting parameters. The results indicate that the fiber orientation angle also has a significant effect on the extent of cutting induced damage and the UD-CFRP workpieces with 0° and 90° present a much better machining mechanism than the ones with 45° and 135°.

Figure 10 shows that the 2-D damage factor F_a_ varies with the fiber orientation under different cutting speed and depth of cut. The F_a_ curve also has four inflection points at the fiber orientation angles of 0°, 45°, 90°, and 135° corresponding to the F_dep_. Nonetheless, F_a_ is not sensitive to the cutting parameters; in the region of 130°–180°, the value of F_a_ is much higher than the rest.

In summary, the 1-D damage factor F_dep_ is a more effective and sensitive index to characterize the cutting induced damage of the machined UD-CFRP with a fiber orientation below 90°; and the 2-D damage factor F_a_ is more suitable for evaluating the extent of damage to the UD-CFRP with a fiber angle more than 90°, because the performance of F_a_ is in good agreement with the phenomenon of the cutting test for the workpiece with the fiber orientation angle of more than 120° that generally has a much worse surface quality and unpredictable damages [11,12,42].

### 4.2. FE Model Validation 

**Figure 11:**
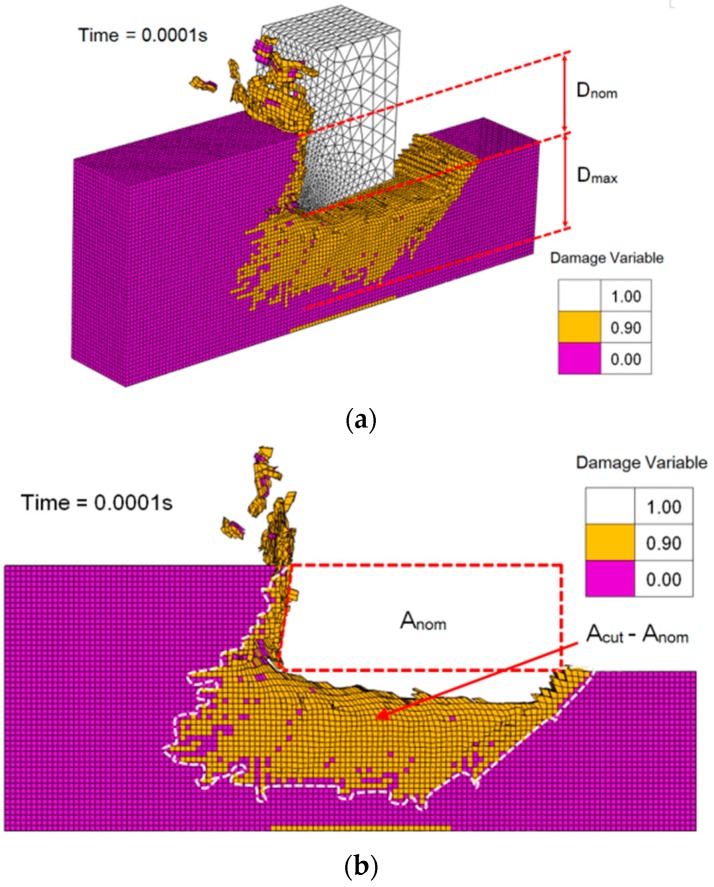
A numerical simulation result of the orthogonal cutting process of a 135° UD-CFRP workpiece when the cutting speed equals 309.5 m/min and the nominal depth of cut equals 0.2 mm: (**a**) Expressions of D_max_ and D_nom_; (**b**) Expressions of A_cut_ and A_nom_.

Figure 11 exhibits the numerical simulation result of the orthogonal cutting process at 0.1 ms while the cutting speed is 309.5 m/min, the depth of cut is 0.2 mm and the fiber orientation is 135°; and expresses the components of damage factors D_max_, D_nom_, A_cut_, and A_nom_. D_max_ is the maximum perpendicular distance between the cutting route and the lowest damaged element. D_nom_ is restricted by the geometric configuration of the FE model. A_nom_ is the area (in 1–2 plane) within the red dashed line in Figure 11b. Also A_cut_ is the sum of the area (in 1–2 plane) within the white dashed line and A_nom_.

To study the cutting induced damage by FEA, the damage variables $d_{f}^{T}$, $d_{f}^{C}$, $d_{m}^{T},$ and $d_{m}^{C}$ required for calculating the damage propagation in Equations (3)–(6) were set as a constant value of 0.9 so that the yellow elements (in Figure 11) could be regarded as damaged elements. With the employment of Python script, the values of D_max_, D_nom_, A_cut_, and A_nom_ were captured by the information of the deleted and damaged elements so that the damage factors F_dep_ and F_a_ could be computed and exported from the 3D numerical model.

**Figure 12:**
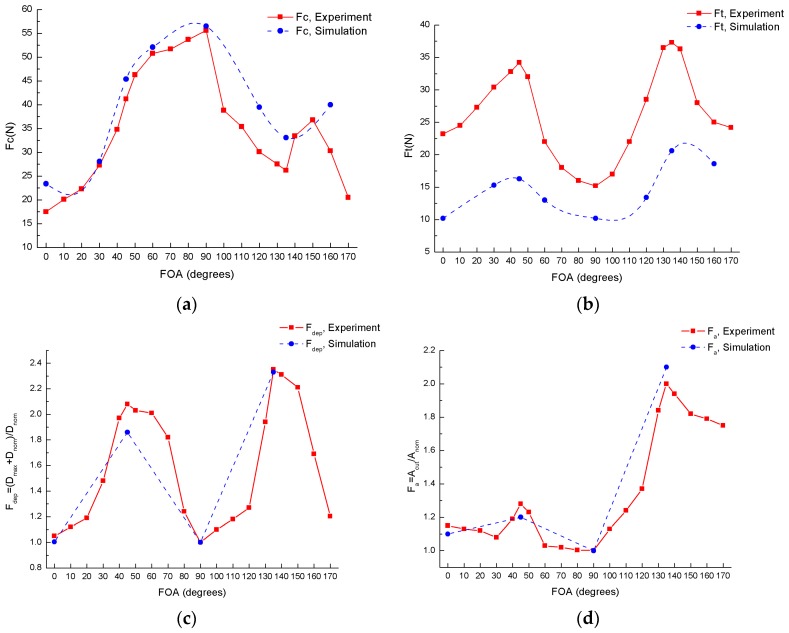
Comparisons between simulation and experimental results with respect to varying fiber orientation angles when cutting speed equals 309.5 m/min (560 r/min) and nominal depth of cut equals 0.2 mm: (**a**) Cutting force F_c_; (**b**) Thrust force F_t_; (**c**) 1-D damage factor F_dep_; (**d**) 2-D damage factor F_a_.

Figure 12 compares the experimental and numerical results of an orthogonal cutting process with a 0.2 mm depth and 309.5 m/min cutting speed. 

The cutting force, thrust force, and damage factor F_dep_ computed through the simulation is in good agreement with the experiments. The simulation model underestimates the influence of the fiber orientation angle on the thrust force. In summary, the FE model is considered to have good compatibility with the orthogonal cutting test and this model is predictable for the cutting forces and damage factors to reduce the cutting induced damage during an orthogonal cutting process. 

### 4.3. Prediction and Optimization of UD-CFRP Orthogonal Cutting Process 

As mentioned above, it can be observed that there are four featured angle values involving 0°, 45°, 90°, and 135° among the whole investigated fiber orientation angles. Within the intervals between the four angles, the relative parameters are changed monotonically so that the parameters with respect to the rest fiber angles can be speculated approximately. 

In the cases of the fiber orientation of 0°, 45°, 90°, and 135°, the validated numerical 3D model of orthogonal cutting was utilized to optimize the cutting parameters to mitigate the cutting induced damage in UD-CFRP laminates.

**Figure 13:**
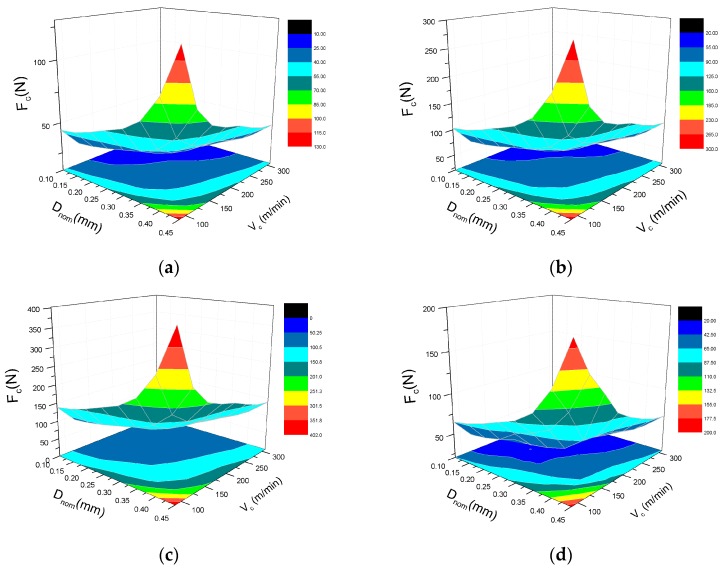
Effect of cutting speed and depth of cut on cutting force F_c_ with the fiber orientation angle of: (**a**) 0°; (**b**) 45°; (**c**) 90°, and (**d**) 135°.

**Figure 14:**
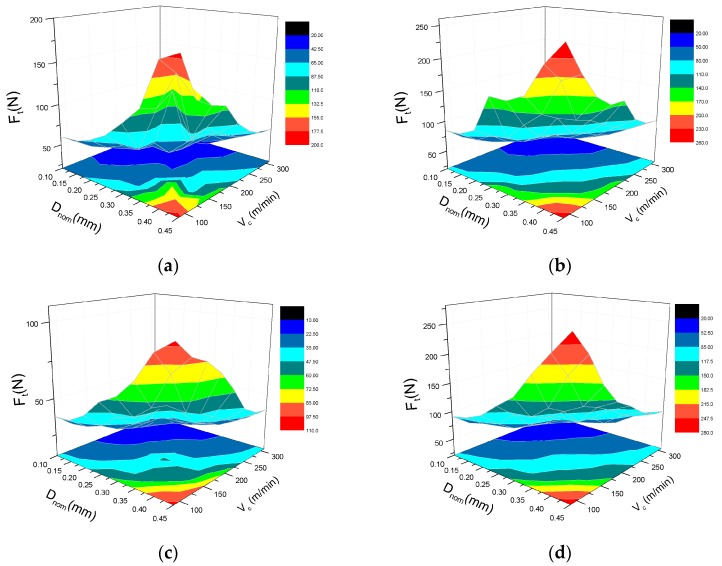
Effect of cutting speed and depth of cut on thrust force F_t_ with the fiber orientation angle of: (**a**) 0°; (**b**) 45°; (**c**) 90°, and (**d**) 135°.

Figure 13 and Figure 14 illustrate that the 3D surface graphs and their projections of the predictions for the cutting force and thrust force are both increased with an increase in depth of cut and decrease in cutting speed. To reduce the energy consumption and improve machining efficiency, the preferential range of cutting parameters is suggested to seriously avoid the big depth of cut of more than 0.3 mm and the low cutting speed of less than 200 m/min.

**Figure 15:**
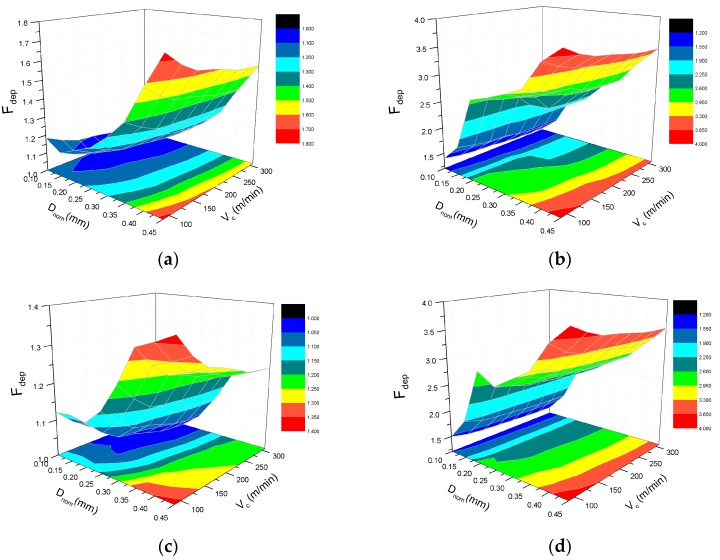
Effect of cutting speed and depth of cut on damage factor F_dep_ with the fiber orientation angle of: (**a**) 0°; (**b**) 45°; (**c**) 90°, and (**d**) 135°.

Figure 15 reveals the effect of cutting parameters on the damage factor F_dep_. The nominal depth of cut significantly influences F_dep_. The extent of cutting induced damage degenerates abruptly when a tiny increment of the depth of cut occurs. However, the cutting speed has a negligible effect on F_dep_ except when the fiber orientation angle is equal to 135°. When the depth of cut is more than about 0.3 mm, the increase of the cutting speed leads to a decline of cutting damage in the case of 135° fiber orientation. F_dep_ varies rapidly with the fiber orientation of 45° and 135°, comparing with 0° and 90°. The cutting induced damaged is more intense when the fiber orientation is either 45° or 135°.

**Figure 16:**
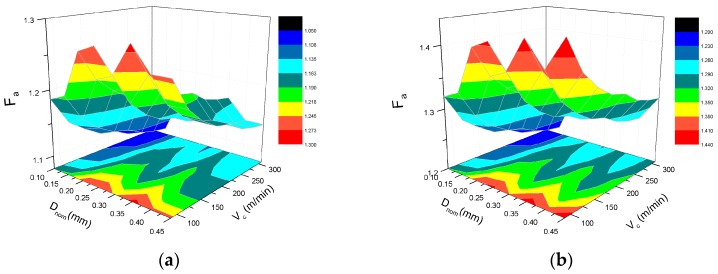

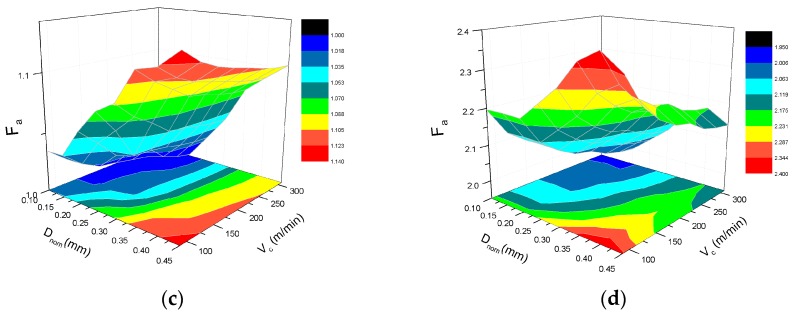
Effect of cutting speed and depth of cut on damage factor F_a_ with the fiber orientation angle of: (**a**) 0°; (**b**) 45°; (**c**) 90°, and (**d**) 135°.

Figure 16 demonstrates the effectiveness of F_a_ to evaluate the cutting induced damage under the condition of different cutting parameters. In Figure 16a,b, the depth of cut seems to have an intricate correlation and a weak influence on the damage factor F_a_, meanwhile the cutting speed has an inversely proportional relationship with F_a_. In Figure 16c, nominal depth of cut has a more forceful effect on F_a_ than the cutting speed. In Figure 16d, it is revealed that there is a synergistic effect of cut depth and cutting speed on the extent of damage.

In summary, to control the extent of cutting induced damage, the optimal cutting parameters of the orthogonal cutting operation of UD-CFRP laminates are advised as a depth of cut of 0.1 mm and a cutting speed of 309.5 m/min. The values of F_c_, F_t_, F_dep_, and F_a_ are presented in Table 4. 

## 5. Conclusions

To study the machining mechanism of the orthogonal cutting process of UD-CFRP, experiments and the finite element method have been both carried out to assess the effect of cutting parameters and fiber orientations on cutting forces and cutting induced damage. In order to quantitatively characterize the extent of cutting induced damage, two types of damage factors are proposed with the assistance of scanning acoustic microscopy and an FE model with 3D Hashin’s criteria and an element-deletion algorithm. The main conclusions are as follow:

1. The FE model has been validated and a good agreement with the experimental data has been found. Furthermore, the optimal cutting parameters have been predicted and the damage factors have been achieved by the model.
2. The fiber orientation angle plays the most crucial role in determining the performance of orthogonal cutting for UD-CFRP, and significantly affects the values of the cutting force, the thrust force, and the cutting induced damage. UD-CFRP has an excellent machined surface quality and internal integrity when the fiber orientation is 0° and 90°.
3. Enhancing the depth of cut can aggravate the cutting damage noticeably, while increasing the cutting speed relieves it. Compared with the cutting speed, the depth of cut definitely has a conspicuous effect on the damage. For an orthogonal cutting process of UD-CFRP, cutting parameters with a low level of the depth of cut and a high level of the cutting speed are suggested.
4. Two damage factors can quantitatively characterize the cutting induced damage by using the experimental and numerical method. Especially, F_dep_ is considered to be the valid and efficient factor in fiber orientation from 0° to 90° and F_a_ is advised to be used in the range of fiber orientation of more than 135°.

## Figures and Tables

**Table 1:** Mechanical properties of T700/TDE-85 unidirectional laminate specimen.

Mechanical Properties		Magnitudes
Young’s modulus in 1–direction	E_11_	138.0 GPa
Young’s modulus in 2–direction	E_22_	10.16 GPa
Young’s modulus in 3–direction	E_33_	10.16 GPa
Poisson’s ratio in 1–2 plane	ν_12_	0.28
Poisson’s ratio in 1–3 plane	ν_13_	0.28
Poisson’s ratio in 2–3 plane	ν_23_	0.30
Shear modulus in 1–2 plane	G_12_	5.86 GPa
Shear modulus in 1–3 plane	G_13_	5.86 GPa
Shear modulus in 2–3 plane	G_23_	4.79 GPa
Density	ρ	1540.0 kg/m^3^
Ultimate tension stress in 1–direction	X_T_	1548 MPa
Ultimate compression stress in 1–direction	X_C_	856 MPa
Ultimate tension stress in 2–direction	Y_T_	37.5 MPa
Ultimate compression stress in 2–direction	Y_C_	218 MPa
Ultimate shear stress in 1–2 plane	S_12_	79 MPa
Ultimate shear stress in 1–3 plane	S_13_	79 MPa
Ultimate shear stress in 2–3 plane	S_23_	60.5 MPa

**Table 2:** Cutting parameters used in the orthogonal cutting test.

Parameters	Units	Range
Cutting speed, V_c_	r/min	160, 200, 250, 320, 400, 450, 500, 560
	m/min	88.4, 110.5, 138.2, 176.8, 221.1, 248.7, 276.3, 309.5
Nominal depth of cut, D_nom_	mm	0.1, 0.15, 0.2, 0.25, 0.3, 0.35, 0.4, 0.45

**Table 3:** Definition of damage factors used for cutting damage quantification.

Category	Damage Factors	Equation	
One-dimensional	F_dep_	(D_max_ + D_nom_)/D_nom_	(1)
Two-dimensional	F_a_	A_cut_/A_nom_	(2)

**Table 4:** Values of parameters with respect to fiber orientation angles under optimal conditions.

Fiber Orientation Angle (°)	Cutting Force, F_c_ (N)	Thrust Force, F_t_ (N)	1-D Damage Factor, F_dep_	2-D Damage Factor, F_a_
0	15.25	20.40	1.06	1.08
45	35.90	28.89	1.23	1.20
90	48.45	12.77	1.003	1.001
135	22.83	30.9	1.33	1.99
